# Effects of Live Combined *Bacillus subtilis* and *Enterococcus faecium* on Gut Microbiota Composition in C57BL/6 Mice and in Humans

**DOI:** 10.3389/fcimb.2022.821662

**Published:** 2022-02-10

**Authors:** Xionge Pi, Weilin Teng, Dibo Fei, Gang Zhao, Wei Liu

**Affiliations:** ^1^ Institute of Plant Protection and Microbiology, Zhejiang Academy of Agriculture Sciences, Hangzhou, China; ^2^ Department of infectious Disease Control and Prevention, HangZhou Center for Disease Control and Prevention, Hangzhou, China

**Keywords:** probiotics, prebiotics, short-chain fatty acids, gut, microbiota

## Abstract

Probiotics, prebiotics, and synbiotics can alleviate metabolic syndrome by altering the composition of the gut microbiota. Live combined *Enterococcus faecium* and *Bacillus subtilis* has been indicated to promote growth and reduce inflammation in animal models. However, the modulatory effects of live combined *B. subtilis* R-179 and *E. faecium* R-026 (LCBE) on human microbiota remain unclear. The current study examined the growth of these two strains in the presence of various oligosaccharides and assessed the effects of this probiotic mixture on human and murine gut microbiota *in vitro* and *in vivo*. Oligosaccharides improved the growth of *E. faecium* R-026 and *B. subtilis* R-179 as well as increased their production of short-chain fatty acids. *E. faecium* R-026 or *B. subtilis* R-179 co-incubated with *Bifidobacterium* and *Clostridium* significantly increased the number of the anaerobic bacteria *Bifidobacterium longum* and *Clostridium butyricum* by *in vitro* fermentation. Moreover, LCBE significantly reduced plasma cholesterol levels in mouse models of hyperlipidemia. LCBE combined with galacto-oligosaccharides led to a significant decrease in the Firmicutes/Bacteroidetes ratio and a significant increase in the relative abundance of *Akkermansia* and *Bifidobacteria* after treating mice with LCBE (0.23 g/day) for eight weeks. Furthermore, *in vitro* fermentation also showed that both the single strains and the two-strain mixture modulated human gut microbiota, resulting in increased *Lactobacillus* and *Bifidobacteria*, and decreased *Escherichia-Shigella*. Overall, these results suggest that LCBE can improve host health by reducing the level of cholesterol in mouse models by modifying the composition of the gut microbiota.

## Introduction

Probiotics are live microorganisms that may confer potential health benefits to hosts when administered orally (Cunningham et al., 2021) and are thought to promote host health through the production of beneficial enzymes, organic acids, vitamins, and nontoxic antibacterial substances. Many studies have reported that probiotics improve host health by modulating the gut microbiota. Some probiotics have been used to treat gastrointestinal diseases including constipation, diarrhea, infection, necrotizing enterocolitis, inflammatory bowel disease, and colon cancer (Liang et al., 2021).

Probiotics can consist of a single bacterial strain or a mixture of strains and can also be combined with prebiotics. Based on a limited number of studies, multi-strain probiotics appear to show greater efficacy than those with single strains. For example, a combination of *Bacillus subtilis* and *Saccharomyces boulardii* was shown to improve digestion, growth, gastrointestinal health, and the overall health status of weaned piglets (Goktas et al., 2021). Similarly, administration of *Bifidobacterium animalis* subsp. *lactis* BB12 with *Enterococcus faecium* L3 significantly ameliorated signs and symptoms of allergic arthritis (Anania et al., 2021).

Several studies have shown that *B. subtilis*, an aerobic bacterium, can utilize a substantial amount of free oxygen in the gut for its proliferation. Dietary supplementation with *B. subtilis* KN-42 has been shown to limit the proliferation of aerobic pathogens and enhance the growth of beneficial anaerobic bacteria such as *Bifidobacterium* and *Lactobacillus*, thus improving the growth performance and digestive tract health of animals (Hu et al., 2014). *Enterococcus faecium* is widely used as a feed probiotic supplement and may have clinical benefits such as suppressing diarrhea as well as improving growth performance and composition of the gut microbiota (Satokari, 2019).

Previous research has shown the beneficial effects of a combined live *B. subtilis* R-179 and *E. faecium* R-026 on host health (Guo et al., 2018; Wang et al., 2021). Live combined *B*. *subtilis* and *E*. *faecium* (LCBE) strains are approved for use as animal feed additives (Jensen and Bjørnvad, 2019). LCBE has been reported to ameliorate colitis in a murine model (Hu et al., 2014), and has shown a protective effect in polymicrobial sepsis through the activation and transformation of macrophages and mast cells (Shi et al., 2017b; Guo et al., 2018). LCBE has also shown to regulate the performance, immune status, and gut microbiota of lactating sows (Huang et al., 2021). Furthermore, administration of LCBE capsules with lactulose for the treatment of functional constipation is being explored in clinical studies (Li et al., 2012). However, the underlying mechanisms of these beneficial effects of LCBE on host health are not well characterized. Moreover, little is known regarding the effects of live combined strains on gut microbiota.

This study aimed to explore the relationship between LCBE and other anaerobic probiotics such as *Bifidobacterium* and *Clostridium*. The potential benefits of LCBE were explored and the ability of the probiotics to utilize prebiotics and their compatibility with other probiotics were assessed.

## Materials and Methods

### Probiotics Strains and Growth Conditions

*B. subtilis* R179 and *E. faecium* R-026 strains were obtained from a commercial probiotic product made by Medilac-S (Beijing Hanmi Pharmaceutical Co. Ltd., China). LCBE contained *E. faecium* (1.35×10^8^ CFU/g) and *B. subitlis* (1.5×10^7^ CFU/g). *B. subtilis* R-179 strains were cultured in Luria-Bertani broth (LB) medium while *E. faecium* R-026 strains were cultured overnight at 37°C in brain heart infusion medium. *Bifidobacterium longum* CECT 7894 and *Clostridium butyricum* MS1 were grown in reinforced clostridial medium (RCM) under anaerobic conditions. Overnight cultures of probiotics strains were used to inoculate the coculture fermentation and batch culture fermentation. RCM medium was used as the coculture fermentation medium of probiotics strains while basic growth medium VI supplemented with 0.8% (*w*/*v*) soluble starch was prepared and used for batch fermentation. Each oligosaccharide as the sole carbon source was dissolved into basic growth medium and autoclaved. The oligosaccharide prebiotics used were: galacto-oligosaccharides (GOS), mannose-oligosaccharides (MOS), isomalto-oligosaccharide (IMO; 95%), fructo-oligosaccharides (FOS; 95%) and resistant dextrin (RDX) purchased from Baolingbao Biological Co. (Shandong, China). Additionally, inulin powder (INU; 95%) was purchased from Fengning Pingan High-tech Industrial Co. Ltd. (China); stachyose powder (STE; 90%) was purchased from Tianmei Biotechnology Co. Ltd. (Xi’an, China); lactulose (LAU) was acquired from Beijing Hanmi Pharmaceutical Co. Ltd. (Beijing, China); raffinose (RAF) was acquired from Aladdin (Shanghai, China); and polyglucan type I (PG1), mannitol (MAI), xylitol (XYI), and sorbitol (SBI) were purchased from Sigma-Aldrich.

### *In Vitro* Coculture and Viable Counts of Probiotic Bacteria

To assess the interaction among four probiotics (*B. fidobacterium, C. butyrium, B. subtilis* R179 and *E. faecium* R-026), four batch experiments were carried on two different strain combinations. The stage and level of bacterial growth were assessed by measuring the optical density of samples using a MicroScreen 16-HT machine (Gering, Tianjin, China). Viable colony counts of anaerobic probiotics were determined using RCM agars in an ElectroTek AW 400TG Anaerobic Workstation (ElectroTek, West Yorkshire, United Kingdom). Four bacterial cultures were grown overnight in RCM medium until they reached the exponential phase. Then, *B. subtilis* R-179 and *E. faecium* R-026 were cocultured with *B. fidobacterium* and *C. butyricum* in a 1:1 ratio (*v*/*v*). Individual bacteria (500 μL) were used as a negative control. The individual bacteria (500 μL) and 1 mL of the two-strain mixture (500 μL of each bacterial strain) were transferred to a 5 mL anaerobic bottle with RCM liquid medium. After 24 h, 1 mL of the cocultures and individual bacteria were removed and homogenized in a 1:10 dilution with phosphate-buffered saline (PBS). Values of colony forming units (CFU)/mL were determined by serial dilution and plating on RCM agar medium. *B. subtilis* and *E. faecium* were streaked on LB medium plates and incubated overnight at 37°C under aerobic conditions. *B. longum* and *C. butyric* were streaked on RCM medium plates and incubated overnight at 37°C under anaerobic conditions. All experiments were independently performed in duplicate.

### Preparation of Probiotics and Experimental Design

Probiotics strains were cultured for 6–8 h in the appropriate media to reach the optimum cell division stage (log phase). The culture was then washed twice using PBS to remove all culture media. Equal counts (1×10^8^ CFU) of newly prepared bacterial cells were pooled in four groups; (1) Control group: human feces slurry only; (2) *B. subtilis* R-179 group: *B. subtilis* R-179 strain with human feces slurry; (3) Enterococci: *E. faecium* R-026 strain with human feces slurry; and (4) Probiotic complex: *B. subtilis* R-179 and *E. faecium* R-026 complex with human feces slurry.

### Measurement of Short-Chain Fatty Acids

Six short-chain fatty acids (SCFA; propionate, acetate, valerate, isobutyrate, isovalerate, and butyrate) were quantified using the gas chromatography technique (Shimadzu, GC-2010 Plus, Japan). Specifically, a DB-FFAP chromatographic column (Agilent, USA) with a hydrogen flame ionization detector was used. Crotonic acid (trans-2-butenoic acid) was used as the internal standard.

### Redox Potential Measurements

After filtration of the fermentation cultures, the redox state of the fermentation supernatant was measured by a redox electrode with an Ag/AgCl reference electrode (Unisense, Aarhus Denmark). The electrode was calibrated with saturated quinhydrone buffer solutions of pH 4 and pH 7 at 30°C. The value of the redox potential was defined relative to the standard hydrogen electrode. Redox data were collected thrice after the detection of baseline stability.

### Animals and Experimental Design

Forty male C57BL/6 mice (eight-week-old) were purchased from Beijing Vital River Laboratory Animal Technology Co. Ltd. (Beijing, China). All mice were housed in an specific pathogen free (SPF) animal room under a controlled environment (temperature: 25 ± 2°C; relative humidity: 50 ± 5%; 12/12 h light/dark cycle). After acclimatization for one week, mice were randomly divided into five groups (n = 8 per group). Except for the control group (N) that received standard chow, the mice were fed a high-fat diet for eight weeks to model hyperlipidemia, which included the model group (M) and three treatment groups (MG, MP, and MPG groups). Mice in the MG group were administered GOS (0.23 g/day) by gavage, mice in the MP group were administered LCBE (0.23 g/day) by gavage, and mice in the MPG group were administered a mixture of equivalent doses of LCBE and GOS dissolved in physiological saline. All chow was purchased from Jiangsu Xietong Biology Co. Ltd. (Nanjing, China). The N and M groups were administered saline (0.1 mL/10 g/day) by gavage. Mice were administered with LCBE and/or GOS and fed the high-fat diet for eight weeks after reaching the hyperlipidemia standard. The average body weight of the model group was 20% higher than the normal group, and the total cholesterol (TC), triglycerides (TG), high-density lipoprotein cholesterol (HDL-C), and low-density lipoprotein cholesterol (LDL-C) levels were significantly increased compared with the control group. At the end of week 8, all mice were fasted overnight and anesthetized with isoflurane. Blood samples were collected from their orbital vein into tubules containing heparin sodium. Fresh feces from each mouse were collected in sterile tubules for gut microbiota analyses. All animal research was approved by the Committee for Animal Ethics of Zhejiang Academy of Agricultural Sciences.

### Analysis of Body Weight Gain and Serum Lipid Levels

Serum levels of TC, TG, HDL-C, and LDL-C in mice were assayed using an automatic biochemical analyzer *Chemray 800* (Rayto Life and Analytical Sciences Co. Ltd.) according to the manufacturer’s instructions.

### Human Gut Microbiota Culture and Fermentation

Ten healthy participants that self-reported no use of antibiotics in at least the three-month period immediately preceding the study were enrolled after providing written informed consent. The study was approved by the Ethics Committee of the Hangzhou Center for Disease Control and Prevention (No. 202047). Gut microbiota was cultured from fresh fecal samples using an automatic fecal system as described previously (Liu et al., 2020). Every sample was diluted with 10% PBS, and a feces slurry was injected into the fermentation chamber. The experiment was divided into the following four groups: control (without probiotics), EF group (supplemented with *E. faecium*), BS group (supplemented with *B. subtilis*), and complex group (supplemented with *E. faecium* and *B. subtilis*). The diluted fecal suspension was made into 10% (*w*/*v*) slurries. Batch fermentation was conducted at 37°C for 24 h using 10% fecal slurry in anaerobic penicillin vials. The cultures were centrifuged and immediately persevered at -80°C for further analysis of microbial community composition.

### DNA Extraction and 16s rRNA Sequencing of Fermentation Samples

Microbial DNA was extracted from 250 mg fecal samples using the Qiagen DNA extraction kit (Qiagen, Germany) following the manufacturer’s protocol. Polymerase chain reaction (PCR) was used to amplify the V3-V4 region of the 16S ribosomal RNA gene, and the primer pair 343F: 5’- TACGGRAGGCAGCAG -3’ and 798R: 5’- AGGGTATCTAATCCT-3’ was used for amplification. The PCR conditions were as follows: initial denaturation for 2 min at 95°C, 20 cycles of denaturation (94°C for 30 sec), annealing (48°C for 30 sec), and extension (72°C for 2 min). The DNA sequencing was conducted on an Illumina MiSeq PE300 system platform operated by Shanghai OE Biotech Technology Co. Ltd. (Shanghai, China).

### Sequence Processing and Bioinformatics Analysis

Sequences were processed by bar-codes using QIIME (version 1.8). Vsearch software with a 97% similarity cutoff was used to remove primer sequences from clean reads as well as to perform clustering to generate operational taxonomic units (OTUs) (Edgar, 2010). The QIIME package was used to select the representative reads of each OTU. All representative reads were annotated, then blasted on the Silva database (version 123; Greengenes) for 16s rDNA using the RDP classifier with a confidence threshold set at 70% (Wang et al., 2007). Mothur was used to calculate alpha diversities, Shannon and Simpson indices, and richness (observed number of OTUs). The Vegan package was used to calculate phylogenetic measures of beta diversity based on genus level abundance profiles. Alpha diversity was calculated by indices of Shannon, Chao1, PD whole tree, Simpson, and observed species. A principal coordinate analysis (PCoA) plot was plotted using the ggplot2 package based on unweighted UniFrac distances. All consensus sequence data of humans and mice were submitted to the National Center for Biotechnology Information Short Read Archive under accession no. SRP233155 and PRJNA753235, respectively.

### Statistical Analysis

Data were presented as mean ± standard deviation. Statistical analyses were performed using SPSS software. Intergroup differences were assessed using one-way analysis of variance, followed by Turkey’s *post hoc* test. *P* values < 0.05 were considered as statistical significance. Linear discriminant analysis (LDA) of effect size (LEfSe) was used to determine the most discriminant taxa and predicted functions (Kyoto Encyclopedia of Genes and Genomes pathways) between the two groups. Wilcoxon rank sum test and LDA analysis were used to measure the LEfSe of the abundant taxon. Two filters, i.e., *P* < 0.05 and LDA score > 2 were used.

## Results

### Coculture of *B. subtilis* R-179 and *E. faecium* With Probiotics and Bacteria Commonly Found in the Gut

**Figure 1:**
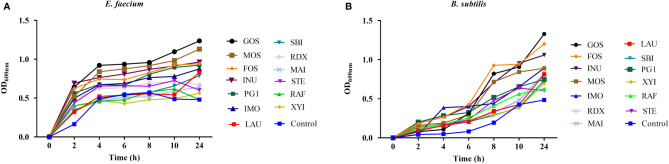
Growth kinetics of *Enterococcus faecium* **(A)** and *Bacillus subtilis* **(B)** when cultured with different oligosaccharides, as measured by optical density (OD) values during 24 h of an incubation period in batch fermentation. basal medium (YCFA), fruto-oligosaccharides (FOS), lactulose (LAU), raffinose (RAF), stachyose (STE), galacto-oligosaccharides (GOS), mannose-oligosaccharides (MOS), isomalto-oligosaccharide (IMO), inulin (INU), polyglucan type 1 (PG1), xylitol (XYI), sorbitol (SBI), mannitol (MAI), resistance dextrin (RDX).

The growth of *E. faecium* R-026 and *B. subtilis* R-179 was examined in the presence of prebiotics commonly found in the gut (**Figure 1**). *E. faecium* R-026 grew rapidly to the exponential phase and reached a stationary phase after 4 h, whereas *B. subtilis* R-179 exhibited a slow increase in growth rate for 24 h. *E. faecium* R-026 showed the most rapid growth in the presence of GOS, followed by MOS, INU, and FOS (**Figure 1A**) compared with the control group. Similarly, *B. subtilis* R-179 (**Figure 1B**) showed the fastest growth in the presence of GOS, FOS, INU, and MOS.

Next, the growth of *E. faecium* R-026 and *B. subtilis* R-179 was examined when cocultured with anaerobic probiotic strains commonly found in gut, i.e., *Bifidobacterium longum* and *Clostridium butyrium.* Both these bacterial strains are under investigation for clinical use. The viability of *E. faecium* R-026 or *B. subtilis* R-179 was not affected by coculture with either *B. longum* or *C. butyricum* (**Table 1**). Additionally, the viability *B. longum* increased from 10.5 ± 0.2 log CFU/mL to 10.7 ± 0.9 log CFU/mL in the presence of *B. subtilis* R-179 and from 10.2 ± 0.2 log CFU/mL to 10.3 ± 0.1 log CFU/mL in the presence of *E. faecium* R-026. Similarly, the viability of *C. butyricum* increased from 2.6 ± 0.2 log CFU/mL to 7.1 ± 0.2 log CFU/mL when cocultured with *B. subtilis* R-179 and from 6.0 ± 1.0 log CFU/mL to 10.4 ± 0.9 log CFU/mL when cocultured with *E. faecium* R-026.

**Table 1:** Viability bacterial counting for individual and mixed cultures of *Enterococcus faecium* or *Bacillus subtilis* cocultured with *Bifidobacterium longum* or *Clostridium butyricum*.

Group	Strains	Viable count (log CFU/mL)	
1		*B. subtilis*	*B. longum*
	*B. subtilis*	9.6 ± 0.1	0
	*B. longum*	0	10.5 ± 0.9
	Mixed culture (*B. subtilis* + *B. longum)*	10.2 ± 0.1	10.7 ± 0.9
2		*B. subtilis*	*C. butyrium*
	*B. subtilis*	10.4 ± 0.9	0
	*C*. *butyrium*	0	7.1 ± 0.2
	Mixed culture (*B. subtilis* + *C. butyrium*)	10.8 ± 0.3	11.4 ± 0.2*
3		*E. faecium*	*B. longum*
	*E. faecium*	10.8 ± 0.2	0
	*B. longum*	0	10.2 ± 0.2
	Mixed culture (*E. faecium* + *B. longum*)	10.2 ± 0.2	10.3 ± 0.1
4		*E. faecium*	*C. butyrium*
	*E. faecium*	10.1 ± 0.2	0
	*C. butyrium*	0	7.1 ± 0.2
	Mixed culture (*E. faecium* + *C. butyrium*)	10.3 ± 0.1	10.4 ± 0.9*

*Indicates significant difference between the mean values within a column (P < 0.05). CFU, colony forming units.

### Production of SCFAs

**Figure 2:**
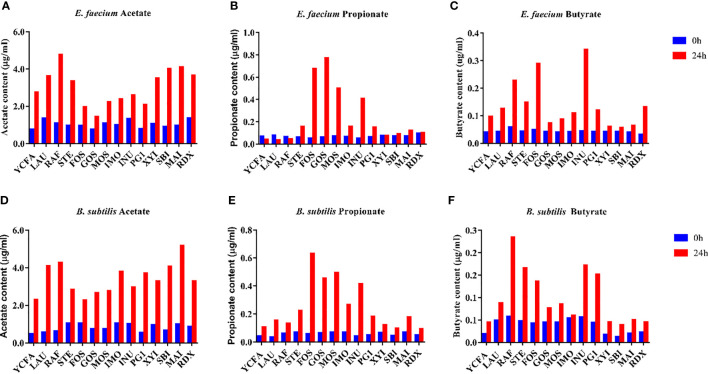
Short-chain fatty acid (SCFA) content after culture of *Enterococcus faecium* **(A–C)** and *Bacillus subtilis* **(D–F)** with different oligosaccharides after 24 h fermentation. basal medium (YCFA), fruto-oligosaccharides (FOS), lactulose (LAU), raffinose (RAF), stachyose (STE), galacto-oligosaccharides (GOS), mannose-oligosaccharides (MOS), isomalto-oligosaccharide (IMO), inulin (INU), polyglucan type 1 (PG1), xylitol (XYI), sorbitol (SBI), mannitol (MAI), resistance dextrin (RDX).

SCFAs produced by gut bacteria are generally thought to be beneficial to the host. Therefore, the production of SCFAs by *E. faecium* R-026 and *B. subtilis* R-179 were examined in the presence of various oligosaccharides (**Figure 2**). After 24 h of culture, both strains were found to have produced a substantial amount of acetate in the presence of RAF, LAU, MAI, and SBI. Similarly, both strains produced a substantial amount of propionate in the presence of GOS, FOS, and MOS. However, butyrate production by both strains was most prominent in the presence of INU, FOS, and RAF. Coculture of *E. faecium* R-026 and *B. subtilis* R-179 produced the highest amount of acetate in the presence of SBI, RAF, and INU, the highest amount of propionate in the presence of MOS, GOS, and IMO, and the highest amount of butyrate in the presence of GOS, PG1, and RDX.

Additionally, SCFA production by *B. subtilis* R-179 or *E. faecium* R-026 was detected after coculture with anaerobic probiotic strains, i.e., *B. longum* or *C. butyricum*. An increase in acetate was more pronounced in the coculture with *B. longum*, while an increase in butyrate was more pronounced in the coculture with *C. butyricum* (**Figure S1**).

### Redox Potential Changes During Probiotic Fermentation

To further understand the effects of *B. subtilis* R-179, *E. faecium* R-026, and their live combination on the chemical environment in the gut, the redox potential was then examined 24 h after culturing the strains anaerobically in a separate or combined system. Lower redox potentials were observed in all cultures; of note, the lowest redox potential was observed in the live combination culture vs cultures of individual strains (**Table 2**).

**Table 2:** Change of redox potential after culture of *Bacillus subtilis* and *Enterococcus faecium* alone and in combination.

Groups	Redox [redox (mv)]
Control group (blank medium)	550.5 ± 4.42^a^
*B. subtilis* group	439.7 ± 7.78^b^
*E. faecium* group	430.7 ± 2.78^b^
Mixed group of *B. subtilis* and *E. faecium*	353.5 ± 1.03^c^

Same letters between the groups indicate no significant difference (P > 0.05), and different letters indicate statistically significant differences (P < 0.05).

### Effects of LCBE on Body Weight and Plasma Lipids

**Figure 3:**
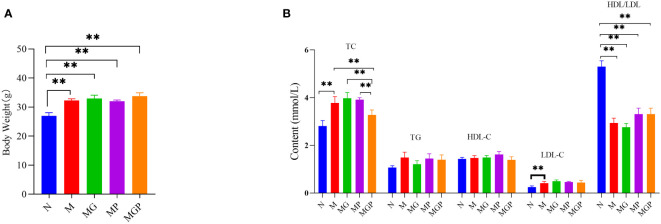
Effects of live culture of *Enterococcus faecium* and *Bacillus subtilis* (LCBE) and galacto-oligosaccharides (GOS) on body weight **(A)** and plasma metabolites **(B)** in mice. N represents the group of mice fed with a standard diet; M represents the group of mice fed with the high-fat diet; MG represents the group of mice fed with the high-fat diet and GOS; MP represents the group of mice fed with the high-fat diet and probiotic mixture; MPG represents the group of mice fed with the high-fat diet, GOS and probiotic mixture. **Indicates significant difference compared with M group (*P* < 0.01).

Next, the potential effects of LCBE were examined using a murine model of obesity. Groups of mice were fed a high-fat diet and given GOS, LCBE, or GOS with LCBE. Mice in all groups fed with the high-fat diet showed a significant increase in body weight compared with mice fed with a standard diet. However, TC levels in mice fed with high-fat diet that received the combination of GOS and LCBE were significantly lower compared with mice fed the high-fat diet alone. There was no significant difference in TC levels between mice on a high-fat diet that received GOS or LCBE alone compared with mice on a high-fat diet alone. No significant differences in TG, HDL-C, or LDL-C levels were noted among the mice on a high-fat diet that received GOS, LCBE, or a combination of GOS and LCBE, and mice on a high-fat diet alone (**Figure 3**).

### Effects of Different Dietary Interventions on Murine Gut Microbiota

The relative abundance of bacterial taxa at the phylum and genus levels were then examined in all five groups of mice, i.e., mice on standard diet, mice on high-fat diet alone, and mice on high-fat diet receiving GOS, LCBE, or a combination of GOS and LCBE. At the phylum level, Firmicutes, Bacteroidetes, Verrucomicrobia, Actinobacteria, and Proteobacteria were the major phyla identified (bacterial proportion >1.0%) among the five groups.

There were significant differences in the ratio of Firmicutes/Bacteriodetes (F/B) between these groups. Compared with mice given standard show, mice fed with the high-fat diet alone showed an increased F/B ratio (0.4 and 1.8, respectively). Treatment with GOS (F/B value= 1.0) or LCBE (F/B value = 1.4) reduced the F/B ratio. Of note, the lowest F/B ratio was noted in mice on a high-fat diet that received the combination of GOS and LCBE (F/B value = 0.3).

**Figure 4:**
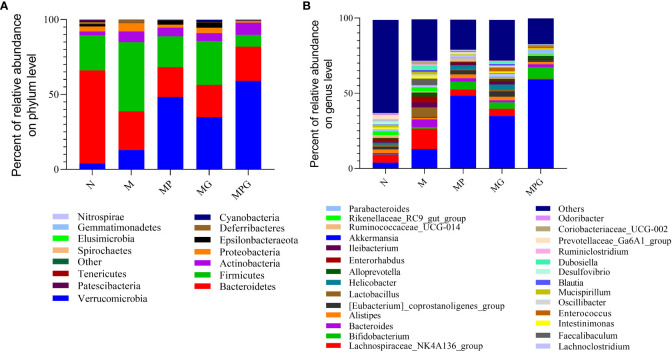
The effect of live culture of *Enterococcus faecium* and *Bacillus subtilis* (LCBE) and galacto-oligosaccharides (GOS) on murine microbial composition. **(A)** Relative abundance of bacteria at phylum level; **(B)** Relative abundance of bacteria at genus level under different treatments. The ordinate represents the species name, the color gradient indicates the proportion of the species.

At the genus level, *Akkermansia* and *Lachnospiraceae_* NK4A136 were the major genera (bacterial proportion >1%) in the gut microbiota of mice. A high-fat diet significantly increased the relative abundance of *Lactobacillus*, *Alloprevotella*, *Faecallibaculum*, and *Enterococcus* compared with mice on a standard diet, and the increased abundance of these genera was attenuated in mice receiving GOS, LCBE, or a combination of GOS and LCBE (**Figure 4B**). Moreover, a significant increase in the abundance of *Akkermansia* and *Bifidobacteria* as well as a decrease in the abundance of *Lachnospiraceae* NK4A136 was noted in mice receiving GOS, LCBE, or a combination of GOS and LCBE compared with mice on a standard diet (**Figure 4A**).

### Effects of *B. subtilis* R-179 and *E. faecium* R-026 on Human Fecal Microbiota

The effects of the probiotic strains on human fecal bacterial communities were compared using a batch fermentation system inoculated with ten human fecal samples. Using high-throughput sequencing, a total of 1,002,166,296 clean reads were obtained from 36 samples after filtering. The value of good coverage in each sample was higher than 0.99. This implies that the 16S rRNA gene from every library represented the largest bacteria proportion. Further analyses found that only *B. subtilis* R-179 treatment improved the diversity of gut microbiota, although no significant differences in alpha-diversity were noted between the control group, the probiotic interventions, and the two-strain combined treatment (**Figure S2**). Moreover, PCoA plots of unweighted UniFrac indicated no clear separation between the control and any of the probiotic treatment groups.

**Figure 5:**
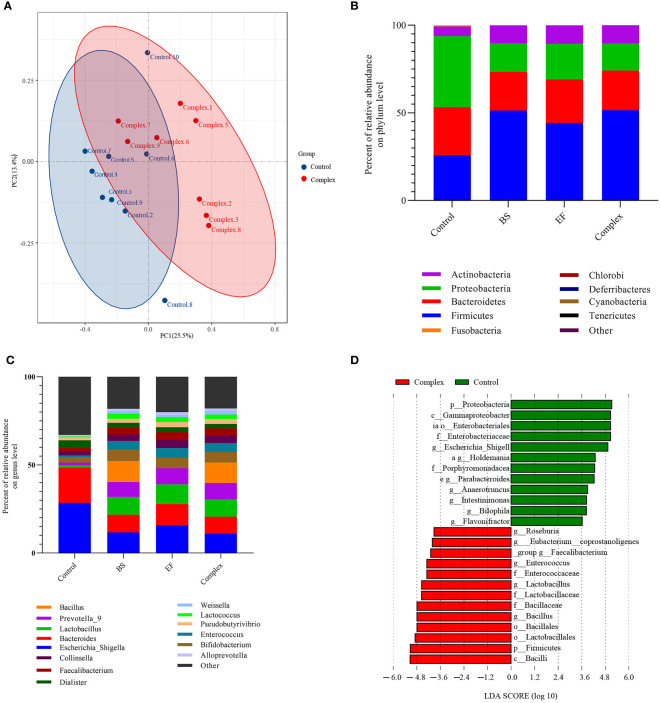
Probiotic treatment differentially changes the human fecal microbiota *in vitro* fermentation after 24 h of anaerobic incubation. **(A)** Principal coordinate analysis (PCoA) of unweighted-UniFrac distances of all human fecal microbiome samples; **(B, C)** Major phyla and genus changes upon *Bacillus subtilis*, *Enterococcus faecium* and probiotic complex treatment over time up to 24 h; **(D)** Microbial cladogram indicating microbial clustering of human fecal microbiota in probiotic complex compared to control treatment. BS represents the *Bacillus subtilis* group, EF represents the *Enterococcus faecium* group.

The bacterial compositions after probiotic treatment in the *in vitro* fermentation systems were analyzed by high-throughput sequencing. The overall microbiota structure at the top 30 phylum level is shown in **Figure 5**. The results revealed that the Proteobacteria phylum was the dominant phylum with the control medium in the absence of probiotics strains. Treatment with *E. faecium* R-026, *B. subtilis* R-179, or LCBE significantly improved the abundance of Actinobacteria and Firmicutes. The population of the Firmicutes phylum increased from 25.94% to 51.40% when cocultured with *B. subtilis* R-179, to 44.25% when cocultured with *E. faecium* R-026, and to 51.57% when cocultured with LCBE. In contrast, a reduction in the abundance of Proteobacter and Fusobacteria phyla was noted.

At the genus level, the abundance of *Bacteroidetes*, *Escherichia-Shigella*, *Bacteroids*, and *Parabacteroides* decreased when cocultured with probiotics (*E. faecium* R-026 alone, *B. subtilis* R-179 alone and LCBE) compared to the control group. Moreover, the abundance of–*Lactobacillus*, *Prevotella_9*, *Bifdobacterium*, *Enterococcus*, *Lactococcus* and *Faecalibacterium* at the genus level were higher when cocultured with probiotics compared to the control group. Abundance of *Bacillus* genus members was also higher when cocultured with *B. subtilis* R-179 or LCBE, although this effect was attributed to *B. subtilis* R-179 with the combined treatment. LEfSe analysis showed that coculture with *B. subtilis* R-179 alone increased *Lactobacillates*, *Lactobacillaceae*, *Lactobacillus*, *Enterococcus*, and *Pseudomonadles*, while coculture with *E. faecium* R-026 alone increased the abundance of *Bacilli*, Firmicutes, *Bacillales*, *Bacillaceae*, *Bacillusensia*, *Eubacterium* c*oprostanoligenes*, and *Bacteroidales* S24-7 (**Figure S3**). In contrast, coculture with LCBE significantly increased *Bacillus*, Firmicutes and *Lactobacillates*, but decreased Proteobacteria, *Enterobacteraceae*, *Gammaprotecobacteria* and *Escherichia-Shigella* populations (**Figure 5**).

SCFAs analyses revealed that coculture with *E. faecium* R-026 alone or LCBE significantly increased the levels of isobutyrate. However, there were no significant alternations in the production of acetate, propionate, butyrate, isovalerate, or valerate in any of the treatment groups (**Table 3**). This indicated that microbiome regulation may increase the production of SCFAs in the gut.

**Table 3:** Short-chain fatty acid (SCFA) concentration of different probiotic treatments after 24 h fermentation.

Probiotics treatment	SCFA (mM)					
	Acetate	Propoinic acid	Butyrate	Isovalentic	Isobutyrate	Valentic
Control group	13.65 ± 7.21	4.01 ± 3.50	0.29 ± 0.19	0.05 ± 0.02	0.04 ± 0.03	0.03 ± 0.04
*Bacillus* group	14.74 ± 6.62	4.91 ± 4.35	0.42 ± 0.22	0.07 ± 0.03	0.06 ± 0.04	0.04 ± 0.05
*Enterococcus* group	11.27 ± 5.88	4.26 ± 4.08	0.08 ± 0.07	0.04 ± 0.04	0.31 ± 0.17^*^	0.08 ± 0.02
Complex group	12.12 ± 5.83	4.61 ± 5.04	0.26 ± 0.21	0.08 ± 0.03	0.21 ± 0.30	0.07 ± 0.07

*Indicates significant difference between mean values within a column (P < 0.05).

## Discussion

A healthy gut microbiota is essential for the well-being of the host. For example, *Bacillus* has been found to promote the production of diverse digestive enzymes and over 45 kinds of antibacterial compounds that suppress the growth of pathogenic bacteria (Peng et al., 2021). *Enterococcus* has been used as a dietary supplement to suppress harmful microorganisms (Chen et al., 2014). *B. subtilis* R-179 and *E. faecium* R-026 have been considered probiotics for a long time (Ben Braïek and Smaoui, 2019; Sun et al., 2019). This study aimed to assess the probiotic effects of *E. faecium* R-026, *B. subtilis* R-179, and LCBE, the live combination of the two strains. The growth pattern of the two strains was first examined and the growth of both strains was found to be accelerated in the presence of oligosaccharides commonly found in the gut. Additionally, the growth pattern of the two strains was not affected by anaerobic strains of bacteria in the gut. Conversely, the growth of anaerobic strains, e.g., *Bifidobacterium* and *Clostridium*, was not affected by the presence of either *E. faecium* R-026 or *B. subtilis* R-179. It was noted that when each strain or LCBE was cultured in the presence of GOS or FOS, the production of butyrate and propionate was significantly increased. These results indicated it is highly likely that *E. faecium* R-026, *B. subtilis* R-179, or the live combination of the two strains may adapt well to the gut microenvironment, and that the addition of these strains to the microbiome may increase the production of SCFAs that are beneficial to the host as well as facilitate the growth of other strains of bacteria that are common in the gut. Of note, it was also found that human fecal bacterial composition was altered after coculture with either strain or LCBE, indicating that the LCBE may modify the micro-environment of the gut *via* modification of the microbiome.

The gut microbiota contributes to the regulation of the chemical environment in the gut. Ben Braïek and Smaoui discovered that oxygen consumption in the colon *via* the process of oxidation is a major cause of antibiotic-triggered dysbiosis of the microbiota (Ben Braïek and Smaoui, 2019). Increased redox potential in the gut may indicate an intestinal inflammatory state or malnutrition. Additionally, antibiotic-induced gut microbiota alterations can disturb the redox dynamics in the gut and result in the overgrowth of facultative anaerobes such as Enterobacteriaceae (Shi et al., 2017a). It was observed in the current study that the redox potential was decreased during the coculture with *E. faecium* R-026 and *B. subtilis* R-179 under anaerobic conditions (**Table 2**), and the results support the hypothesis that redox dynamics can be altered by a specific bacterial taxon present within the intestinal microbiota.

In addition, LCBE combined with GOS was shown to attenuate an increase in TC levels in the plasma; however, no significant differences in TG, HDL-C, or LDL-C levels were observed in mice fed with a high-fat diet. In the current study, an increase in the F/B ratio was noted in mice on a high-fat diet due to an increased abundance of Firmicutes and decreased abundance of Bacteroidetes. This phenomenon has been reported to contribute to the pathogenesis of colonic inflammation in patients with inflammatory bowel disease and other functional gastrointestinal disorders (Walker et al., 2011). Administration of LCBE with GOS in mice on a high-fat diet attenuated this increase, and it is highly likely that the combination of LCBE and GOS promoted the growth of *Bifidobacterium*. This observation is consistent with previous reports that demonstrated the important role of the *Bifidobacterium* population in preventing the development of obesity and insulin resistance (Seganfredo et al., 2017). Similarly, at the genus level, mice administered with both LCBE and GOS showed an increased abundance of the genera *Lachnospiraceae* NK4A136, a strain that has been reported to improve host health (Stadlbauer et al., 2020). Moreover, the reduced abundance of the genus *Dialister* was observed, a strain associated with IBS and spondyloarthritis (Tito et al., 2017; Lopetuso et al., 2018). Therefore, LCBE may promote the growth of beneficial bacteria and suppress the growth of harmful bacteria in the gut.

The use of probiotic bacterial strains has been explored in clinical studies for the treatment or prevention of diarrhea and hepatopathy. Among these, *B. subtilis* R-179 has shown some potential as a treatment for oral candidiasis due to its ability to inhibit the growth and proliferation of *Candida* spp. (Zhao et al., 2016). Moreover, a live combination of *B. subtilis* R-179 and *E. faecium* R-026 has been used as a probiotic supplementation to inhibit the growth of *Heliobacter pylori* (Stein, 2005). Additionally, *B. subtilis* R-179 and *E. faecium* R-026 can also restore the entire fecal microbiota after the use of antibiotics and thus play a role in ameliorating secondary infection in clinical patients (Lu et al., 2014). The current study indicated that LCBE may adapt well to the gut microenvironment and promote SCFA production and the growth of other bacterial strains that are beneficial to the host. Furthermore, LCBE conferred some benefits to mice on a high-fat diet and may modify the composition of the gut microbiota, and thereby provide further health benefits to the host. Further studies are required to confirm the potential benefits of LCBE on host health.

## Data Availability Statement

The raw data supporting the conclusions of this article will be made available by the authors, without undue reservation.

## Ethics Statement

The research was approved by the Committee for Animal Ethics of Zhejiang Academy of Agricultural Sciences.

## Author Contributions

WL, GZ, and XP designed the study, performed data analysis, and wrote the manuscript. WL and XP conducted the experiments. DF and WT collected all fecal samples. All authors read and approved the final version of this manuscript.

## Conflict of Interest

The authors declare that the research was conducted in the absence of any commercial or financial relationships that could be construed as a potential conflict of interest.

## Publisher’s Note

All claims expressed in this article are solely those of the authors and do not necessarily represent those of their affiliated organizations, or those of the publisher, the editors and the reviewers. Any product that may be evaluated in this article, or claim that may be made by its manufacturer, is not guaranteed or endorsed by the publisher.
