# Supplementary material for: Effects of Live Combined Bacillus subtilis and Enterococcus faecium on Gut Microbiota Composition in C57BL/6 Mice and in Humans
Source: Front Cell Infect Microbiol. 2022 Feb 10;12:821662. doi: 10.3389/fcimb.2022.821662 (PMC8866766; doi:10.3389/fcimb.2022.821662)
Supplement: Supplementary file 1 [file DataSheet_1.pdf]

Supplementary materials

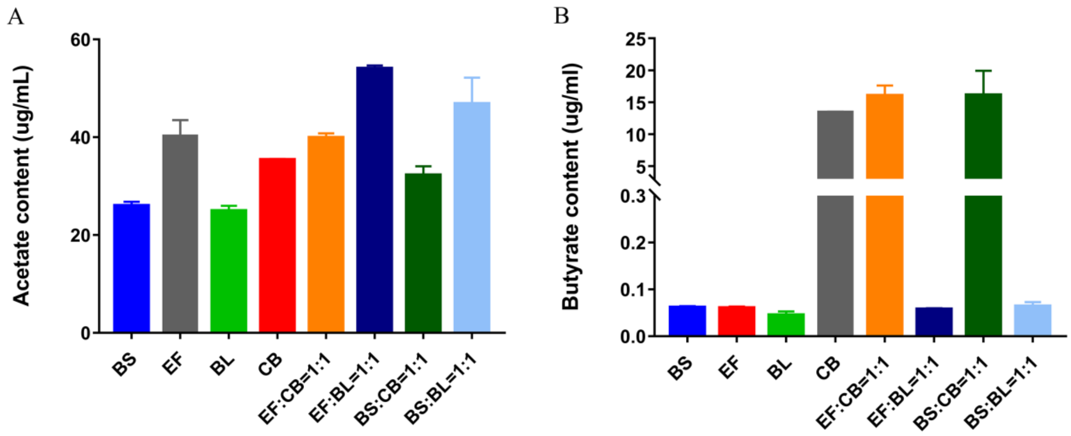

Figure S1. The concentration of acetate (A) and butyrate (B) of *B. subtilis* and *E. faecium* co-cultured with *B. longum* and *C. butyricum* respectively for 24 h *in-vitro* fermentation.

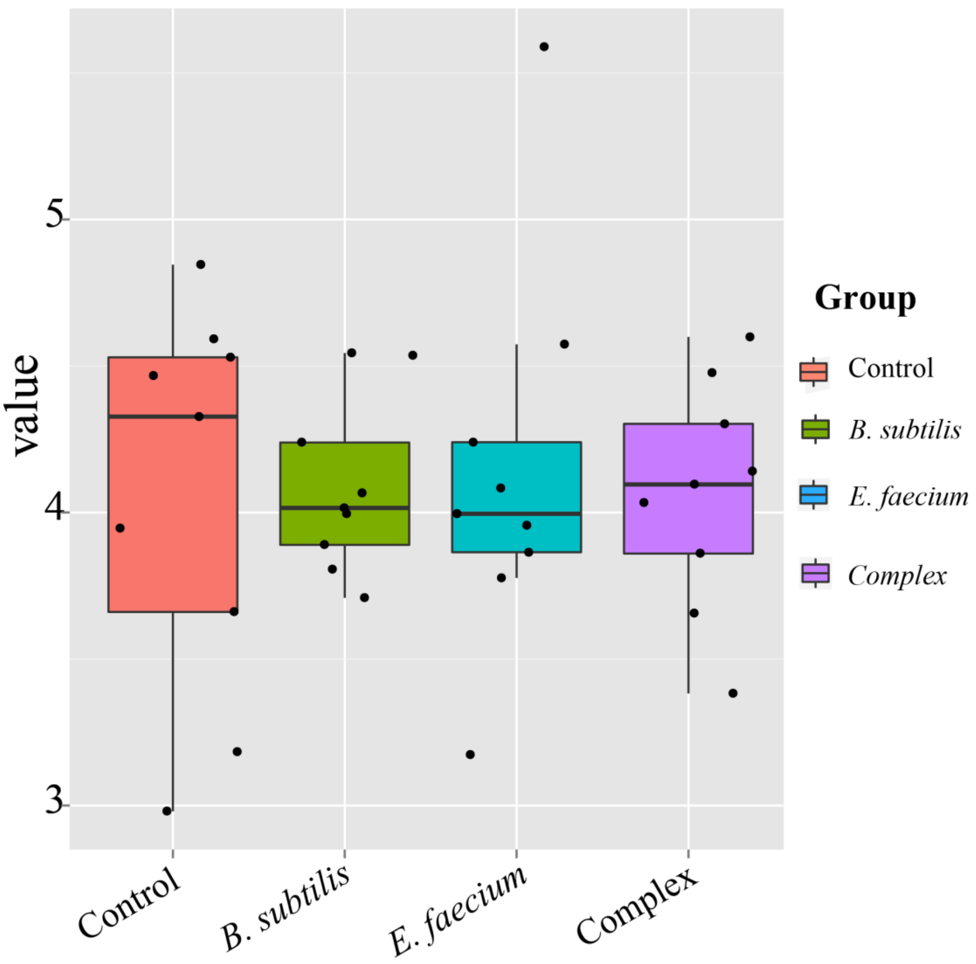

Figures S2. Shannon index in *Bacillus*, *Enterococcus* and probiotics complex treatment.

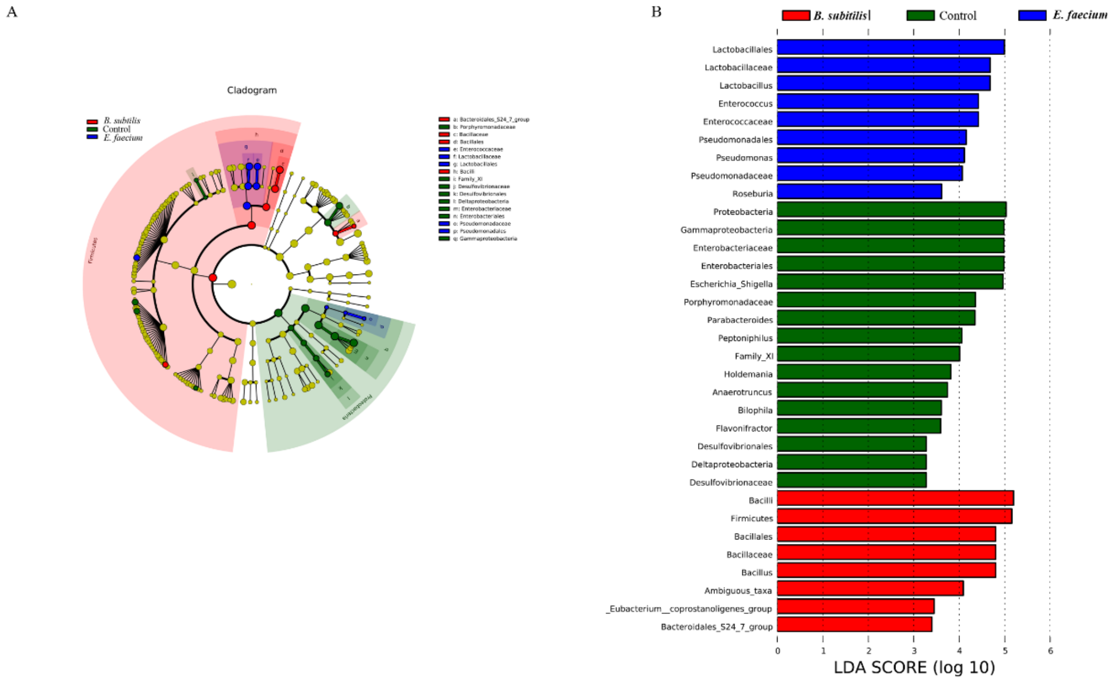

Figure S3. Microbial cladogram indicating microbial clustering of human fecal microbiome in *Bacillus subtilis*, *Enterococcus faecium* compared to control treatment.
